# Bone Marrow Mononuclear Cell Transplantation Restores Inflammatory Balance of Cytokines after ST Segment Elevation Myocardial Infarction

**DOI:** 10.1371/journal.pone.0145094

**Published:** 2015-12-21

**Authors:** Kirsi Alestalo, Johanna A. Miettinen, Olli Vuolteenaho, Heikki Huikuri, Petri Lehenkari

**Affiliations:** 1 Surgery Clinic, Medical Research Center, Oulu University Hospital, Oulu, Finland; 2 Department of Anatomy and Cell Biology, Medical Research Center, University of Oulu, Oulu, Finland; 3 Medical Research Center Oulu, Oulu University Hospital and University of Oulu, Oulu, Finland; 4 Department of Physiology, Institute of Biomedicine, University of Oulu, Oulu, Finland; University of Kansas Medical Center, UNITED STATES

## Abstract

**Background:**

Acute myocardial infarction (AMI) launches an inflammatory response and a repair process to compensate cardiac function. During this process, the balance between proinflammatory and anti-inflammatory cytokines is important for optimal cardiac repair. Stem cell transplantation after AMI improves tissue repair and increases the ventricular ejection fraction. Here, we studied in detail the acute effect of bone marrow mononuclear cell (BMMNC) transplantation on proinflammatory and anti-inflammatory cytokines in patients with ST segment elevation myocardial infarction (STEMI).

**Methods:**

Patients with STEMI treated with thrombolysis followed by percutaneous coronary intervention (PCI) were randomly assigned to receive either BMMNC or saline as an intracoronary injection. Cardiac function was evaluated by left ventricle angiogram during the PCI and again after 6 months. The concentrations of 27 cytokines were measured from plasma samples up to 4 days after the PCI and the intracoronary injection.

**Results:**

Twenty-six patients (control group, n = 12; BMMNC group, n = 14) from the previously reported FINCELL study (n = 80) were included to this study. At day 2, the change in the proinflammatory cytokines correlated with the change in the anti-inflammatory cytokines in both groups (Kendall’s tau, control 0.6; BMMNC 0.7). At day 4, the correlation had completely disappeared in the control group but was preserved in the BMMNC group (Kendall’s tau, control 0.3; BMMNC 0.7).

**Conclusions:**

BMMNC transplantation is associated with preserved balance between pro- and anti-inflammatory cytokines after STEMI in PCI-treated patients. This may partly explain the favorable effect of stem cell transplantation after AMI.

## Introduction

The heart has limited anaerobic metabolism, and myocytes are dependent on oxygen and a constant flow of nutrients. In acute myocardial infarction (AMI), the ischemic event launches cellular trauma, an intense inflammatory response, and a remodelling process, all of which contribute to changes in ventricular geometry and stiffness [1]. The cytokines recruit neutrophils and macrophages, contribute to the release of other cytokines and growth factors, and are the key regulators of the inflammatory phase. Eventually, ventricular remodelling has an impact on cardiac contraction ability and the development of heart failure.

Myocardial repair is orchestrated by the controlled release of proinflammatory cytokines, which enhance the inflammation process [2,3]. The increases of interleukin (IL)-1ra and tumor necrosis factor alpha (TNF-α) are associated with cell apoptosis and decreased ejection fraction (EF) after AMI [4,5]. It is anticipated that simultaneous or subsequent release of the anti-inflammatory cytokines reduces and limits the inflammation process [3]. Consequently, balance between the proinflammatory and anti-inflammatory cytokines is crucial for optimal cardiac repair. The equilibrium between the proinflammatory cytokines, such as IL-1ra, IL-1β, IL-6, TNF-α, interferon-γ (IFN-γ), and anti-inflammatory cytokines, such as IL-4, IL-10, and IL-13, is often skewed one way or the other in various diseases. Stem cell therapy is under intensive research to treat several diseases, especially where their immunomodulatory capability is essential (Table 1). Clinical and preclinical studies show that stem cell transplantation after AMI increases EF and there is supporting data for tissue repair, though the overall results are still controversial [6–9]. The cytokines secreted by mesenchymal stem cells (MSCs) are thought to modulate the inflammation process by contributing to the recruitment and secretion of inflammatory cells, modulating the remodelling process, and promoting cell survival and tissue repair [10]. Dayan V. et al showed enhancement of cardiac function and the MSC-mediated secretion of IL-10 after AMI and MSC transplantation [11]. In addition, some of the favorable effect of MSC transplantation on myocardial repair may be an outcome of modulation of macrophage phenotype and function [12]. MSC transplantation increases percentage of reparative M2 macrophages, that also might affect on the remodeling and scar size.

**Table 1:** BMMNC and mesenchymal stem cells in various diseases.

Disease	Effect of transplantation	Mechanism	Reference
Cardiac infarction	Enhancement of cardiac function	MSC-mediated secretion of IL-10	[11]
Cardiac infarction	Reduced infarction size, improved LV function,	Enhancement of angiogenetic factors such as VEGF, reduction of apoptosis	[32]
Cardiac infarction	Improved LVEF, increase in angiogenesis	CXCR-4-SDF-1	[33]
Cardiac infarction	Decreased LV dilatation and dysfunction	Attenuated IL-6 and TNFα production Increased expression of IL-10	[17]
Cardiac infarction	Increased fractional shortening and LVEF	VEGF	[34]
Cardiac infarction	Infarct healing	Modulation of macrophage phenotype → IL-10, IL-6, TNFα	[35]
Cardiac infarction	Improved LV function	Increased IL-10	[18]
Cardiac infarction	Collagen formation	IL-6	[36]
Cardiac infarction	Survival after AMI	low IL-6 level	[37]
Cardiac infarction	Collateral remodeling	VEGF	[38]
Type 1 diabetes mellitus	Repair the destroyed islets in diabetic mice	MSC differentiation and immunomodulatory effects	[39]
Arthritis	Decrease in cartilage erosion	IL-4, IL-10, IFNγ	[40]
Arthritis	Improved arthritis symptoms	Decrease in TNFα and inflammatory cells, Increased expressions of anti-inflammatory cytokines (IL-10, IP-10 and CXCR3)	[41]
Traumatic Brain Injury	Improvement in neurological function	Reduction of inflammatory cells and proinflammatory cytokines, increase in anti-inflammatory cytokines	[42]
Abdominal Aortic Aneurysm formation	Attenuated formation	IL-17 production	[43]
Spinal cord compression Injury	Promotes tissue sparing	NGF	[44]
Acute lung injury	Reduced collagen deposition and neutrophilic infiltration	Decrease in pro-inflammatory cytokines (IL-1β, IL-6, TNFα) and VEGF, nitrate and nitrite.	[45]
Acute kidney injury	Improved kidney function	IL-1β, IL-6, TNFα, IL-4,	[46]
Cerebral ischemia	Decrease in neuronal apoptosis and improved neurological function	Increase in IL-10 expression	[47]

In our previous double-blinded clinical study, we observed increased EF in ST segment elevation myocardial infarction (STEMI) patients treated with bone marrow mononuclear cell (BMMNC) transplantation after thrombolysis and percutaneous coronary intervention [PCI] [13]. In addition, no differences were observed in the adverse clinical events, arrhythmia risk variables, or restenosis of the stented coronary lesions. A more marked improvement of EF after intracoronary infusion of BMMNCs appears in patients with the most severe impairment of EF on admission [14]. In our pre-clinical study, the number of transplanted BMMNC in myocardium seem to associate with the improvement of EF after AMI [15]. However, we were unable to describe the exact mechanism underlying the better outcome. Our objective was to study the effect of BMMNC transplantation on the cytokine network and the inflammation process that contributes to remodelling after AMI. The purpose of this study was to reveal changes in separate anti-inflammatory or proinflammatory cytokine levels that would correlate with the improved EF. The previous studies reveal that BMMNC or MSC transplantation increases the level of anti-inflammatory cytokines, such as IL-10 [16–18]. Our preliminary hypothesis was that these anti-inflammatory cytokines would dominate in the BMMNC group and at least partially explain the biological effect. However, this simplified hypothesis was proven wrong and here, we propose an alternative explanation for the improved outcome observed in these patients.

## Materials and Methods

### Patients

The data was collected in Finland, at the University Hospital of Oulu or the University Hospital of Turku, between October 2001 and February 2007. A total of 522 consecutive patients with STEMI treated with intravenous thrombolytic therapy were screened for the original study [13]. Inclusion criteria were: age younger than 75 years, STEMI confirmed with troponin release and electrocardiography, thrombolytic therapy initiated within 12 hours of symptoms, no cardiogenic shock, no rescue PCI due to chest pain and no need for urgent PCI immediately after thrombosis, no need for coronary bypass graft surgery, hemodynamic instability, no refusal of the patient to participate, and no severe coexisting condition that interfered with the patient’s ability to comply with the protocol. Exclusion criteria are described in the previous study [14]. The majority of excluded patients did not meet inclusion criteria (n = 368). A minority refused to participate (n = 32) or had other reasons (n = 42). Participants provided written informed consent to participate in the study within 2 days after thrombolytic therapy. The study protocol was approved by the Ethical Committee of the Northern Ostrobothnia Hospital District and was conformed to the Declaration of Helsinki.

### Study design

**Fig 1:**
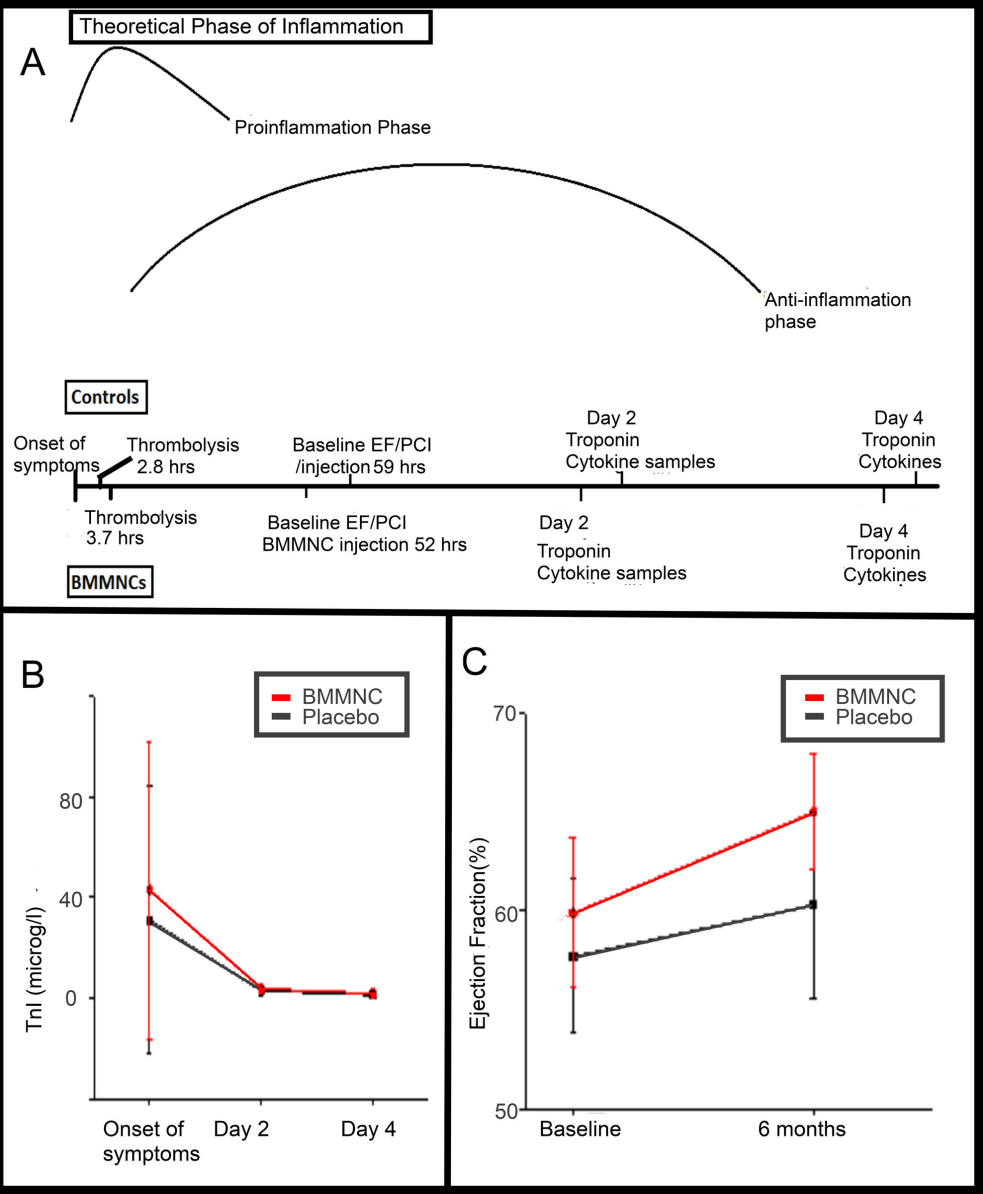
Study design and signs of STEMI. A) Thrombolysis was performed during proinflammatory reaction launched by ischemic condition. PCI and injection followed 40–77 hours (mean time presented in the figure) after onset of symptoms and cytokine sampling was performed 2 and 4 days after that. B) Troponin level were measured immediately after onset of symptoms and 2 and 4 days later. As a sign of STEMI, the levels increased similarly in both study groups but declined 2 days later. No statistically significant differences existed (control N = 12; BMMNC n = 14; p = 0.71). C) Contrast opacification of left ventricular angiograms was used to analyse EF at the baseline and after 6 months to evaluate effect of BMMNC transplantation on cardiac function. There was grater absolute increase in EF in BMMNC treated patients. However, the difference was not statistically significant possibly due to small sample size (control n = 11; BMMNC n = 13; p = 0.15).

This was a double-blinded study in which the patients were randomly assigned in a 1:1 ratio to have either BMMNC or placebo treatment (Fig 1A) [13]. Bone marrow aspiration, cell collection, and cell preparation were performed 40–77 hours after the onset of symptoms. PCI was considered as a baseline time point.

### Cell isolation, preparation, and administration

Under local anaesthesia, 80 mL of bone marrow was aspirated from the posterior iliac crest into a heparin-treated syringe to harvest mononuclear cells from the bone marrow on the morning of the PCI day. The bone marrow aspirate was subjected to density-gradient centrifugation on Ficoll-Hypaque to exclude granulocytes and erythrocytes. After the mononuclear cells were collected from the interphase, they were washed twice with heparinized physiological saline. Mononuclear cells were suspended in 10 mL of medium containing 5 mL of the patient’s own serum and heparinized physiological saline. The BMMNC suspension was filtered and quality-control procedures (i.e., microbial culture for sterility and flow cytometer analysis to count CD34+ cells) were performed. After the BMMNC separation procedure, the cells were administered by intracoronary injection. The placebo medium contained physiological saline. The validity of the cell preparation system was assessed as described previously [13].

The culprit coronary lesion supplying the infarct area was recognized, and PCI was performed using the standard techniques with implantation of paclitaxel drug-eluting stents for all patients. After stenting, medium containing BMMNCs or placebo medium was administered by intracoronary injection over the wire balloon using intermittent balloon inflation in the stent.

### Measurement of the left ventricular ejection fraction

To evaluate cardiac function, the left heart ventricular angiography was performed at baseline with catheterization with PCI, and was repeated identically 6 months later (Fig 1A). The left ventricular angiograms were analysed using the Philips Integris BH5000 system (Philips Medical System Netherland B.V., The Netherlands). The left ventricular volume and left ventricular ejection fraction (LVEF) were calculated using the biplane area-length method; the left ventricular outflow tract was included in the measurements [19].

### Biochemical analysis

Blood sampling was performed 0–10 days after the onset of symptoms. The mean time to PCI and injection (i.e., baseline sampling time) was 56 hours after STEMI, and the final sample was taken at the control visit 6 months after the index event. The serum was stored at -20°C until cytokine analysis. The plasma samples were collected in ethylenediamine tetra-acidic acid tubes on ice and immediately spun down, and the plasma was stored at -70°C until analysis. An Innotrac Aio! analyser (Innotrac Diagnostics, Turku, Finland) was used to determine the concentration of troponin-I (TnI) from plasma samples (Fig 1A).

### Cytokines

The serum samples from baseline, 2 days post-PCI, and 4 days post-PCI were used in the cytokine measurements (Fig 1A). The cytokines and related proteins (IL-1-beta, IL-1 receptor antagonist (IL-1ra], IL-2, IL-4, IL-5, IL-6, IL-7, IL-8, IL-9, IL-10, IL-12, IL-13, IL-15, IL-17, eotaxin, basic fibroblast growth factor (FGF), granulocyte colony stimulating factor (G-CSF), granulocyte-macrophage colony stimulating factor (Gm-CSF), IFN-γ, IP-10, MCP-1, MIP-1-alpha, MIP-1-beta, platelet-derived growth factor (PDGF-BB), RANTES, TNF-α, and VEGF) were quantified with the Bio-Rad Bio-Plex Pro Human Cytokine Grp I Panel (27-plex) using a Luminex MagPix system and Luminex xPonent Software (data in S1 and S2 Datasets). The serum samples were diluted fourfold and assayed in duplicate. Milliplex Analyst software (VigeneTech) was used for the multiplex assay data extraction. The coefficient of determination (R^2^) of the 5-parameter logistic regression standard curves was between 0.998 and 1.000 in all assays. For each analyte, the assay working range was determined using the Milliplex software on the basis of intra-assay precision (%CV) and standard curve recovery.

### Cytokine balance

The balance between anti-inflammatory and proinflammatory cytokines was measured to evaluate the effect of BMMNC transplantation on the inflammation process after AMI. The sum of the concentrations of the proinflammatory cytokines (IL-1ra, IL-1β, IL-6, TNF-α, IFNγ) and anti-inflammatory cytokines (IL-4, IL-10, IL-13) was calculated at baseline, 2 days post-PCI, and 4 days post-PCI. The percentage change from baseline to 2 days post-PCI and from baseline to 4 days post-PCI was measured, and Kendall’s tau was used to evaluate the correlation between the changes in anti-inflammatory cytokines and proinflammatory cytokines.

### Statistical analysis

Statistical analysis was performed using SPSS SmartViewer version 22.0 (SPSS, Inc.). The Shapiro-Wilk test was used to test normality. Student’s *t*-test or the Mann-Whitney U test was used to assess the distribution of variables between the study groups. P-values less than 0.05 were considered statistically significant. Due to skew distribution and rather small sample size, Kendall’s tau was used to measure correlation between two rates; a value of 0.6 was considered a significant finding.

## Results

### Patient characteristics

This is a sub-study of the previously reported FINCELL study, in which 522 patients were assessed for eligibility and 80 patients were included the trial [13]. For this sub-study, equal time delay from onset of symptoms to PCI and the injection was the only inclusion criteria for patients included from the original study (control group, n = 12; BMMNC group, n = 14; total n = 26). Table 2 provides a detailed characterisation of the patients. The mean age of the patients was 59 ±13 years in the control group and 60±10 years in the BMMNC group. The mean time delay from the symptoms to PCI was 56 hours (control group, mean 59±10; BMMNC group, 52±12). The two study groups were well matched regarding characteristics at baseline.

**Table 2:** Characteristics of patients.

	Control mean ±SD (n = 12)	BMMNC mean ±SD (n = 14)
Age	59 ±13	60 ±10
Male gender	10	13
Previous Infarct (n)	0	1
Diabetes mellitus (n)	0	4
Time delay to thrombolysis (h)	3.7 ±4.4	2.8 ±2.7
delay Time from symptoms to PCI (h)	59 ±10	52 ±12
Troponin 2 days after AMI (μg/L)	3.0 ±2.0	1.9 ±0.6
**Severity of CAD**		
One-vessel	8	6
Two-vessel	3	5
Three-vessel	1	3
**Number of injected BMCs**		
Number of mononuclear cells (x10^6^)		461 ±130
Number of CD34+ cells (x10^6^)		2.8 ±1.7
**Medication at discharge (n)**		
Aspirin	12	13
Betablocker	9	9
Clopidogrel	1	3
Statin	12	14
Diuretic	3	4
ACE-inhibitor/ATII recaptor blocker	8	12
**Medication at 6 month follow-up (n)**		
Aspirin	12	14
Betablocker	12	12
Clopidogrel	12	12
Statin	12	14
Diuretic	1	3
ACE-inhibitor/ATII recaptor blocker	8	10
M**ajor complications (n)**	0	0
Before discharge from hospital		

### Troponin release

The TnI concentration in the plasma samples was determined at 2 and 4 days after the onset of symptoms (Fig 1A). At 2 days, mean TnI concentration increased in both study groups (mean TnI control group, 3.0 SD 2.0; BMMNC group mean TnI, 3.3 SD 1.9) (Fig 1B). The difference between the groups was not statistically significant (p = 0.71).

### Ejection fraction

Left ventricular angiograms were available for the 11 control group patients and 13 BMMNC group patients at baseline and at 6 months (Fig 1A). The mean absolute change of the global LVEF was higher in the BMMNC group (placebo 1.1 SD 10.2 vs. BMMNC 9.5 SD 16.0) (Fig 1C). In comparison to the previous study, due to a patient dropout rate and high variation, the difference was not statistically significant (p = 0.15).

### Cytokine results

**Fig 2:**
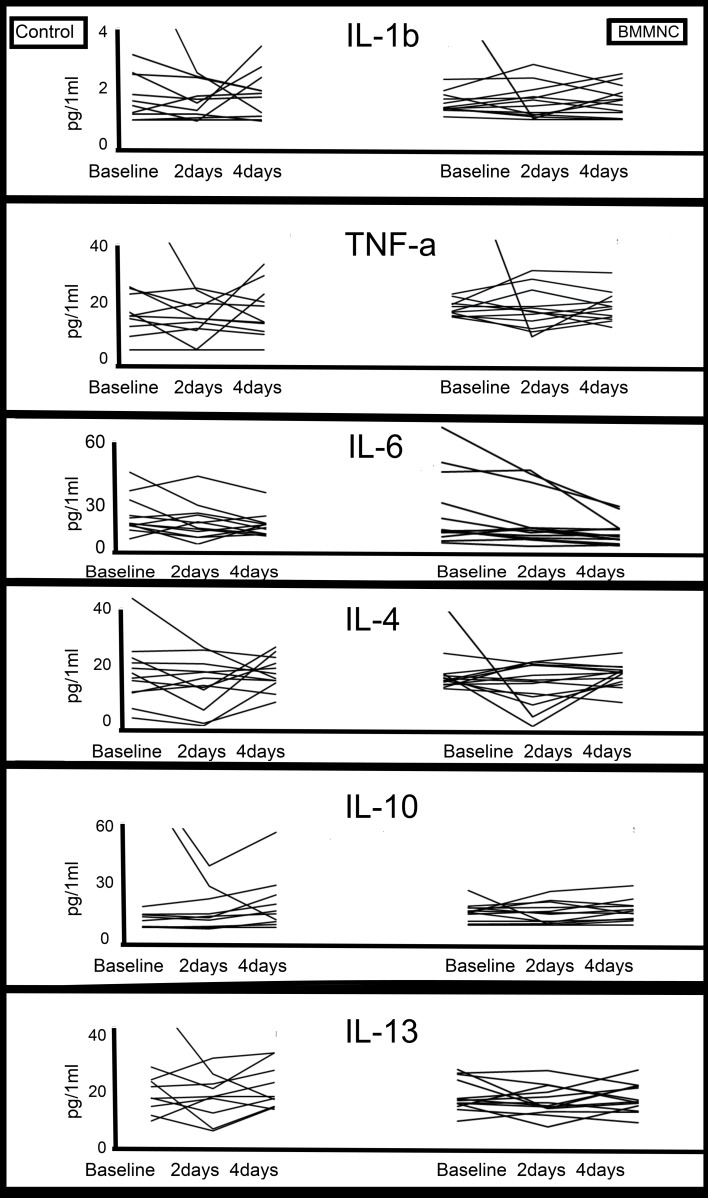
Cytokine levels of single patients. Due to high variability among cytokine concentrations of the single patients, no clear trend in levels existed in either study groups. This illustrates the complexity of cytokine network and systemic factors contributing on inflammation process and cardiac repair.

To test our preliminary hypothesis, cytokine concentrations were measured at baseline, as well as 2 and 4 days after PCI and injection (Fig 1A). At baseline, the cytokine levels in the study groups did not differ significantly (Table 3). Absolute concentrations of the proinflammatory cytokines IL-6, TNF-α, and IL-1b in individual patients are shown in Fig 2. The baseline values were measured immediately after PCI and injection (Table 3). Proinflammatory cytokine levels varied widely among the patients. At 2 days after PCI and injection, IL-6 levels varied from 1.12 to 11.54 pg/mL in the control group and from 0.96 to 11.42 pg/mL in the BMMNC group.

**Table 3:** Cytokine Concentrations at baseline.

	Control mean ±SD	BMMNC mean ±SD	P-value
IL-1b^†^	0.5 ±0.2	0.5 ±0.4	0.60
IL-1ra^†^	35.3 ±47.3	21.7 ±24.2	0.38
IL-2^†^	4.9 ±6.6	2.7 ±2.2	0.27
IL-4^†^	4.5 ±2.7	4. ±1.9	0.09
IL-5^†^	1.9 ±1.2	1.7 ±0.9	0.56
IL-6^†^	4.9 ±3.2	5.7 ±4.7	0.58
IL-7^†^	2.4 ±2.1	1.9 ±0.9	0.42
IL-8^†^	6.0 ±5.0	4.9 ±3.6	0.53
IL-9^†^	2.7 ±2.1	2.5 ±1.9	0.83
IL-10^†^	11.9 ±22.7	2.6 ±8.4	0.13
IL-12^†^	25.3 ±32.3	11.0 ±5.3	0.11
IL-13^†^	6.6 ±7.2	3.9 ±1.2	0.18
IL-15^†^	6.1 ±0.9	6.9 ±3.1	0.37
IL-17^†^	2.4 ±2.1	1.9 ±0.9	0.29
Eotaxin	15.6 ±10.6	16.7 ±9.6	0.77
Fibroblast growth factor (FGF)basic^†^	8.4 ±14.3	4.5 ±5.8	0.35
Granulocyte growth stimulating factor (G-CSF) ^†^	14.5 ±13.3	12.2 ±8.4	0.59
Granulocyte-macrophage colony stimulating factor (Gm-CSF) ^†^	11.8 ±1.2	11.8 ±1.2	1.00
Interferon-γ (IFNγ)^†^	17.3 ±25.5	13.3 ±19.4	0.65
IP-10	140.9 ±37.7	151.1 ±59.1	0.61
MCP-1/MCAF^†^	9.6 ±8.7	12.0 ±14.7	0.62
MIP-1a^†^	1.7 ±1.7	1.5 ±1.0	0.69
MIP-1β	18.1 ±10.6	15.8 ±5.7	0.47
RANTES*	21081.0 ±9016.9	22284.6 ±6016.4	0.68
Tumor necrose factor-α (TNF-α)^†^	5.7 ±4.1	5.7 ±5.0	0.99
Vascular endothelial growth factor (VEGF)^†^	48.0 ±102.1	23.3 ±15.8	0.38

^†^ = concentrations under the threshold of the standard line were included

* = concentrations over the threshold of the standard line were included

Similarly high variability was observed regarding anti-inflammatory cytokine levels (Fig 2). IL-10 and IL-13 concentrations exhibited a trend toward high variability in the control group, while modest variability was measured among BMMNC-treated patients. At 2 days after PCI and injection, IL-10 concentrations varied from 0.82 to 82.5 pg/mL in the control group and from 0.99 to 5.76 pg/mL in the BMMNC group; at 4 days after PCI and injection, IL-13 concentration varied from 2.9 to 22.55 and from 1.89 to 6.07 pg/mL, respectively. In summary, we observed broad variation in baseline cytokine levels, and were unable to confirm any visible or statistically traceable trend between the groups.

**Fig 3:**
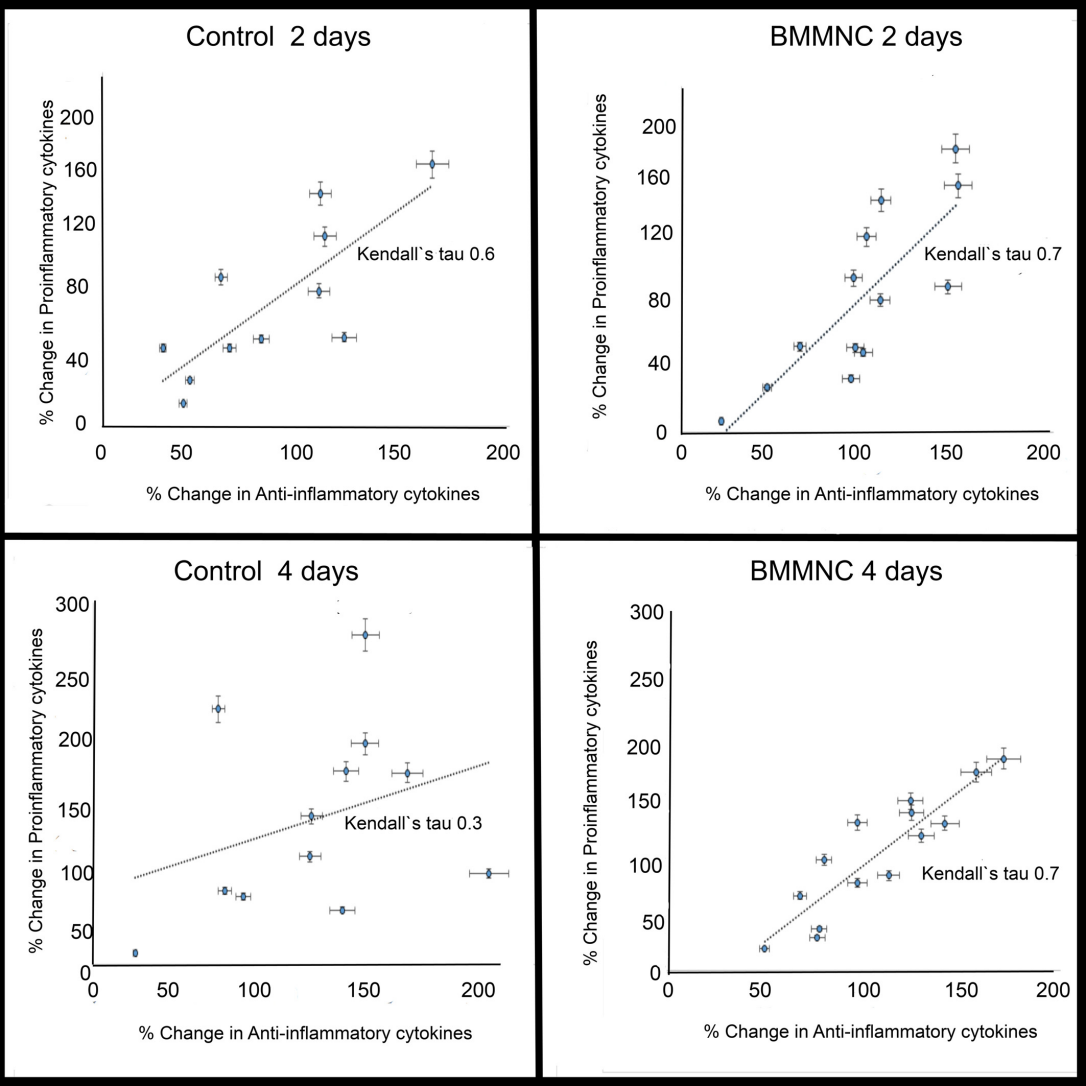
Balance in inflammatory cytokines. The percentage change of proinflammatory cytokines (IL-6, IL-1β, IL-1ra, IFN-γ, TNF-α) and anti-inflammatory cytokines (IL-4, IL-10, IL-13) was measured between time point baseline to 2 days and baseline to 4 days. Change in proinflammatory cytokines correlated with the change in anti-inflammatory cytokines in both study groups at 2 days. At 4 days, correlation remained only in BMMNC treated patients showing restored balance in inflammatory process.

We also measured the correlation of percentage changes in anti-inflammatory cytokines (IL-4, IL-10, IL-13) and in pro-inflammatory cytokines (IL-6, TNF-α, IL-1b, IL1-ra, IFNγ) from baseline to 2 days after PCI and injection and from baseline to 4 days after PCI and injection (Fig 3). We observed a correlation in the percentage change of the anti-inflammatory and pro-inflammatory cytokine concentrations in both study groups at 2 days after PCI and injection (placebo group, Kendall’s tau 0.6, p = 0.01; BMMNC, Kendall’s tau 0.7, p = 0.001). At 4 days after PCI and injection, there was a clear correlation between anti-inflammatory and pro-inflammatory cytokines in the BMMNC group (Kendall’s tau 0.7, p<0.001) but not in the control group (Kendall`s tau 0.3, p = 0.17).

## Discussion

Our initial aim was to evaluate the changes in cytokine levels as a possible mechanism leading to improved EF in patients with thrombolysis and PCI-treated STEMI after BMMNC transplantation. The study groups were relatively equal with respect to demographic and clinical parameters, diseases, and medication at discharge and at 6 months after STEMI. Increases in the troponin level did not differ significantly, indicating that the groups had similar infarction damage. Indeed, the ejection fraction was improved in the BMMNC group, although the difference was not statistically significant after the exclusion of patients from whom we did not obtain reliable serum samples at all time points. To examine our initial hypothesis, levels of a wide variety of cytokines were measured to analyze the acute inflammatory reaction.

In our earlier study, we did not detect any significant association between IL-6 and changes in EF in BMMNC-treated or placebo patients [14]. We thought that measuring the entire cytokine spectrum might reveal some difference in the cytokine network contributing to cardiac repair mediated by the remodeling process. Our data clearly shows that the balance between anti- and proinflammatory cytokine levels was very similar in both groups during the initial phase; however, at 4 days after PCI and injection, BMMNC transplantation influenced this balance favorably (Fig 3). There was a clear correlation between the percentage change in anti- inflammatory and proinflammatory cytokines levels in BMMNC-treated patients at 4 days after PCI and cell transplantation. Ability to modulate inflammation is one possible explanation for the improved cardiac repair and function shown in previous studies [20].

The proinflammatory phase following AMI is necessary and plays a crucial role in ventricular remodeling and proper healing [21]. A prolonged proinflammatory phase leads to adverse remodeling and ventricular dysfunction [10]. The proinflammatory reaction and the switch to the anti-inflammatory phase happen hours after AMI [22]. Consequently, all cytokine samples in this study were measured during the anti-inflammatory phase (Fig 1A). The attenuated levels of proinflammatory cytokines (IL-6, IL-1β, and TNFα) and increased expression of anti-inflammatory cytokine IL-10 after AMI and MSC transplantation have been demonstrated in previous studies [20]. In this study, we were unable to confirm any statistically traceable trend in cytokine levels between the groups.

In the present study, we performed BMMNC transplantation via an intracoronary route. Due to difficulty of sampling, the cytokine concentrations were measured in blood samples obtained from systemic vein. It should also be kept in mind that few cells administered by intracoronary transplant remain in the myocardium, and the transplanted cells affect other organs in addition to the myocardium [15,23]. Our results indicate that BMMNC transplantation after STEMI affects the balance between proinflammatory and anti-inflammatory cytokines, which acutely modulates the global immune system. The capability for immunomodulation enables extensive opportunities for stem cell therapy, because a wide spectrum of diseases is associated with changes in cytokine regulation (Table 1) [24]. A detailed understanding of the immunomodulation effect of stem cells is crucial for optimal repair and stem cell therapy. This study only reveals a correlation between proinflammatory and anti-inflammatory cytokine levels after BMMNC transplantation. Additional studies are required to fully understand the cytokine network and how it is affected by stem cell transplantation.

Inflammatory M1 macrophages dominate at the early phase of infarction following abundance of M2 reparative macrophages on day 7 after injury. Ben-Mardechai et al. showed that MSC injection favours M2 polarization[12]. These macrophages secrete anti-inflammatory cytokines such as IL-10 in order to prevent an over-expansion of the inflammatory cytokines [25]. Cytokines secreted by macrophages may play a role also in our study results and is one possible mechanism for stem cells to balance the inflammation process.

One limitation of this study is the relatively small number of patients. As a result, the increase in EF in the BMMNC-treated patients was not statistically significant, even though our previous study (which included more patients) demonstrated a marked increase. Another limitation is the variation in time delay from onset of symptoms to PCI, which determines the baseline time point and additional cytokine samples; this might explain the different initial cytokine levels and other unknown variables, such as infectious status. Baseline sample collection times varied from 40 to 77 hours from the onset of symptoms, and cytokine measurements were performed at 2 days and at 4 days after baseline (baseline control group, mean 59±10; BMMNC group, 52±12) (Fig 1A). These shortcomings are mostly unavoidable, but should be considered when the data is interpreted. Altogether, the initial 2–4 days only depict the acute phase. Another study with more participants should be conducted to assess any long-term immunomodulatory effect.

Our aim was to study the effect of BMMNC transplantation on proinflammatory and anti-inflammatory cytokines in STEMI patients. We measured the concentrations of IL-4, IL-10, and IL-13 to explore the anti-inflammation process after AMI. The rationale for choosing these anti-inflammatory cytokines was that all selected cytokines have been shown to reduce the inflammatory process in many models and conditions [26–28]. The levels of IL-1ra, IL-1β, IL-6, TNF-α, and IFNγ were measured to illustrate the proinflammatory reaction. Expression of TNFα, IL-1β, IFNγ, and IL-6 are consistently increased after AMI, promoting inflammatory injury as a result of leukocyte recruitment and chemokine synthesis [29,30]. IL-1Ra is a problematic cytokine; as a pure receptor antagonist of IL-1β, it should act as an anti-inflammatory cytokine [31]. However, IL-1β is not readily secreted to the systemic circulation, and level determinations are unreliable in plasma. IL-1Ra has this property, and its production is increased by the same stimuli as IL-1β; therefore, it is a reliable surrogate marker for the action of IL-1β.

Our object was to study mechanism leading for improvement in cardiac function in STEMI patients treated with BMMNC transplantation. We conclude that BMMNCs have a balancing effect on inflammation process that has a crucial role in remodeling and cardiac repair after myocardial infarction. This study demonstrates a correlation between the anti-inflammatory and proinflammatory cytokines in BMMNC-treated STEMI patients at 4 days. Stem cell transplantation has a smaller effect than anticipated on individual cytokines, but is capable of restoring the balance in the inflammatory process. This might partly explain the favorable effect of bone marrow cell injection on cardiac repair.

## Conclusions

Intracoronary BMMNC transplantation during PCI for STEMI patients influences systemic cytokine levels by maintaining the balance between proinflammatory and anti-inflammatory cytokines.

## Supporting Information

S1 Dataset Cytokine raw data. (XLSX)

S2 Dataset Proinflammatory and anti-inflammatory cytokine data. (XLSX)

## Abbreviations

AMI: Acute myocardial infarction
BMMNC: Bone marrow mononuclear cell
EF: Ejection fraction
FGF: Fibroblast growth factor
GM-CSF: Granulocyte-macrophage colony stimulating factor
G-CSF: Granulocyte colony stimulating factor
IFN-γ: Interferon-γ
IL: Interleukin
LVEF: Left ventricular ejection fraction
MSC: Mesenchymal stem cell
PCI: Percutaneous coronary intervention
PDGF-bb: Platelet-derived growth factor
STEMI: ST segment elevation myocardial infarction
TNF-α: Tumor necrosis factor alpha
TnI: Troponin-I
